# Pulmonary artery diameter: means and normal limits—assessment by computed tomography angiography

**DOI:** 10.1093/icvts/ivab308

**Published:** 2021-11-17

**Authors:** Tim Berger, Matthias Siepe, Björn Simon, Friedhelm Beyersdorf, Zehang Chen, Stoyan Kondov, Christopher L Schlett, Fabian Bamberg, Aleksandre Tarkhnishvili, Salome Chikvatia, Martin Czerny, Bartosz Rylski, Maximilian Kreibich

**Affiliations:** 1 Department of Cardiovascular Surgery, University Heart Centre Freiburg, Freiburg, Germany; 2 Faculty of Medicine, Albert-Ludwigs-University of Freiburg, Freiburg, Germany; 3 Department for Diagnostic and Interventional Radiology, Medical Centre-University of Freiburg, Freiburg, Germany; 4 Department of Cardiology and Angiology II, University Heart Centre Freiburg, Freiburg, Germany

**Keywords:** Pulmonary artery, Pulmonary artery aneurysm, Normal pulmonary artery diameter

## Abstract

**OBJECTIVES:**

Normal pulmonary artery (PA) diameter remains blurred and the definitions of PA aneurysm are heterogenous. We aimed to assess PA diameters, identify a threshold for normal diameters, define PA aneurysms, possible predictors of PA size and evaluate the correlation with mid-ascending aortic diameters.

**METHODS:**

Between April 2018 and August 2019, 497 consecutive patients who underwent whole-body computed tomographic angiography were reviewed. Clinical and imaging data were collected from our institutional database. Precise three-dimensional centreline measurements were taken. Linear regression analysis was performed to detect parameters associated with PA diameter. A two-stage model was created to identify potential predictors and the resulting statistically significant interactions were tested. Data were grouped and PA, standard deviation, and upper normal limits were calculated.

**RESULTS:**

Among 497 patients with an average age of 51.4 (20.2) (74.6% males), the mean PA diameter measured 32.0 (4.6) mm [female: 31.2 (4.7) mm vs male: 32.2 (4.5) mm; *P* = 0.032]. The mean PA length, left PA and right PA diameters were similar between male and female patients. We found a significant correlation (*r* = 0.352; *P* < 0.001) between the PAs and mid-ascending aortic diameters. Body surface area (*P* = 0.032, β =  4.52 [0.40; 8.64] 95% CI) was the only significant influencing variable for PA diameter.

**CONCLUSIONS:**

The normal mean PA diameter in a reference cohort is 32.0 (4.6) mm. Body surface area is the only influencing variable of PA diameter. The normal diameters measured and corresponding upper limits of normal revealed that a PA aneurysm should not be considered below a threshold of 45 mm.

**Figure:**
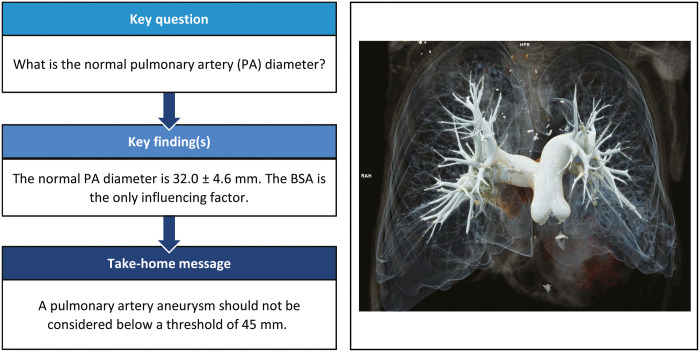

## INTRODUCTION

Pulmonary artery aneurysms (PAA) are rare, and their incidence is low [1]. PAA is usually diagnosed in patients with congenital heart disease, but they also occur in patients with connective-tissue disorders or acquired vascular diseases (infections, vasculitis) and are associated with pulmonary artery hypertension (PAH) [2–5]. But to distinguish a normal pulmonary artery (PA) size from pathological diameters, it is essential to define the standard values. There are a few small studies and data to date from the Framingham Heart Study with considerable limitations related to study size, the accuracy of measurement methods in a single two-dimensional axial slice and image quality [3, 4, 6, 7]. Moreover, the definition of a PAA is very heterogeneous [8–10]. Table 1 illustrates the variety of PAA definitions in published studies.

**Table 1:** Previous pulmonary artery aneurysm definitions

Author	Year	PAA definition	Reference
Nguyen ET *et al.*	2007	PA: >29 mm, right PA: >17 mm	[9]
Chetty KG *et al.*	1996	>4 cm	[11]
Brown JR and Plotnick G	2008	>4 cm	[8]
Veldtman GR *et al.*	2003	Giant PAA >5 cm	[12]
Reisenauer J *et al.*	2017	Large aneurysm: 5–8 cm, giant aneurysm >8 cm	[13]
Duijnhouwer AL *et al.*	2016	1.5-Fold the normal diameter	[10]

PA: pulmonary artery; PAA: pulmonary artery aneurysm.

The aim of this study was to assess PA diameters, identify a threshold for normal diameters and define PAA. We also aimed to identify possible risk factors and influencing predictors of PA size and evaluate any correlation with mid-ascending aortic diameters in high-quality computed tomography (CT) scans.

## PATIENTS AND METHODS

IRB approval was obtained on 27 February 2020 by the institutional review board of the University of Freiburg (# 71/20). Informed consent was waived due to our analysis’ retrospective nature. There was no patient and public involvement regarding analysis,

### Patients and data collection

Seven hundred and eighteen consecutive patients underwent whole-body CT for suspected trauma injuries at the Emergency Department of the University Hospital Freiburg, Germany, between April 2018 and August 2019.

Clinical and imaging data were collected retrospectively from our institutional database. Patients were excluded for the following reasons: non-contrast-enhanced scans (*n* = 15), age <18 years (*n* = 26), pulmonary embolism (*n* = 1), pulmonary fibrosis (*n* = 4), tension pneumothorax (*n* = 1), acute aortic syndrome (*n* = 6), poor quality scans (*n* = 5), extra-corporal life support (*n* = 3) and previous aortic surgery (*n* = 1) to reduce the bias of contributing diseases on PA diameter. The clinical database was reviewed for demographic data and cardiovascular risk factors, which revealed missing data on an additional 159 patients. Accordingly, our final cohort comprises 497 patients for careful analysis. Patient selection is shown in Supplementary Material, Fig. S1.

### CT scan analysis and measurements

A standardized ECG-gated contrast-enhanced whole-body CT scan was taken in each patient via second-generation dual-source CT (Somatom Definition Flash, Siemens Healthineers, Erlangen, Germany) following the split bolus technique application protocol for contrast agents (region of interest in the ascending aorta).

**Figure 1:**
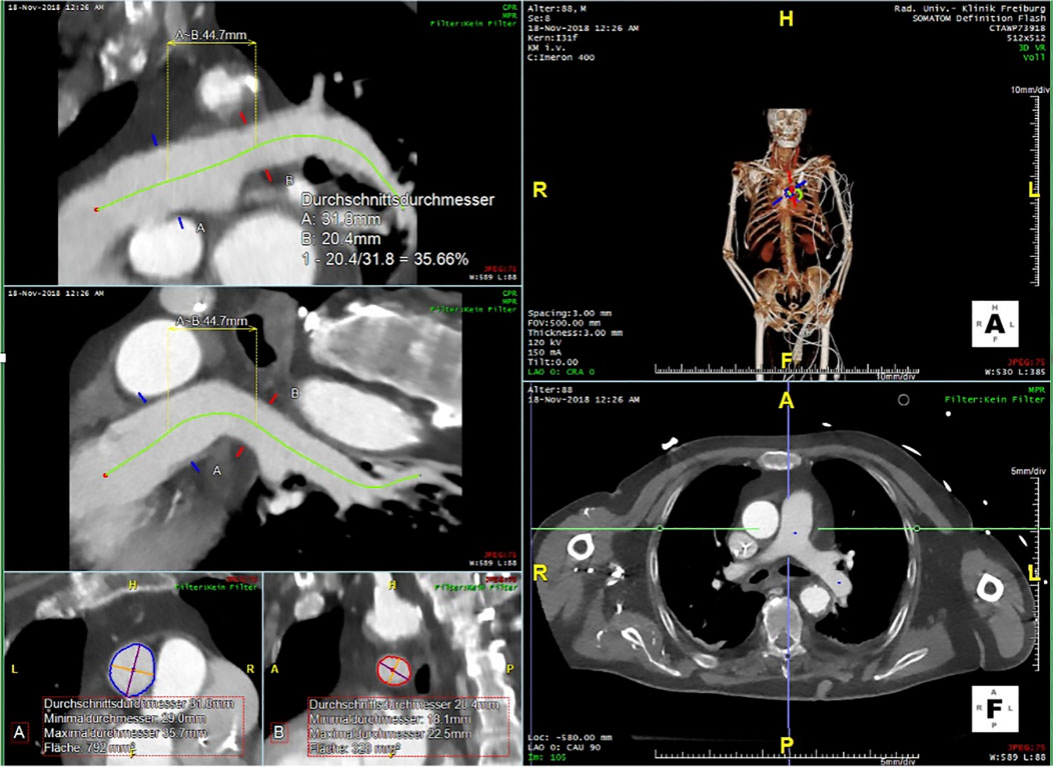
Centreline measurement using the software’s CPR tool. Measurement of the corresponding diameter and area were provided.

CT images were transferred to imaging software (Aquarius iNtuition, TeraRecon Inc, USA) for precise three-dimensional measurements using the software’s CPR tool. The centreline of the pulmonar(Color online) y trunk and PA was defined automatically and adjusted manually if necessary. Diameter and surface area (mm^2^) of the pulmonary trunk and right and left PA were calculated subsequently. The length of the pulmonary trunk was measured from the pulmonary valve to the PA bifurcation. We analysed the ascending aorta’s length from the aortic valve to brachiocephalic trunk and its largest diameter, as well as the surface within that segment. Detailed stepwise measurements are shown in Fig. 1.

### Statistical analysis

Binary and continuous baseline patient characteristics were summarized as number (%) and mean [standard deviation (SD)], respectively. We refrained to test PA diameter for normality as normality can be assumed for large thoracic vessels. To assess the difference between male and female patients, we performed Pearson’s Chi-squared tests on binary variables and the Student’s *t*-tests on continuous variables. Afterwards, we calculated the mean, SD and normal upper limit (mean + 2 SD) for maximum PA length, and the diameters of left PA, right PA and PA. We then summarized the PA characteristics after stratifying the patients by age and BMI. Lastly, we created an exploratory model to identify possible predictors for pulmonary trunk diameter from a two-stage, stepwise, backward selection process, with significance levels of 0.10. At stage 1, we started with a full linear regression model containing all potential influencing factors, including age (categorical), sex, body surface area (BSA) (m^2^ as continuous variable), BMI (kg/m^2^), smoking history, diabetes mellitus, dyslipidaemia, hypertension and coronary artery disease. Subsequently, at stage 2, we began selecting by specifying a full model with all main effectors, i.e. remaining variables from the stage 1 selection, as well as all possible pairwise interaction terms between the main effectors. The sum of cardiovascular risk factors was included as continuous variable. Robust variance estimators were used in all regression steps [14]. All analyses were exploratory in nature. As a result, *P*-values and 95% confidence intervals were not corrected for multiple comparisons and inferences drawn from them may not be reproducible. A PA diameter index was established by calculating PA diameter/BSA. Sum of cardiovascular risk factors was defined as the amount of cardiovascular risk factors that include smoking history, diabetes mellitus, dyslipidaemia, hypertension and coronary artery disease. Statistical analyses were performed using IBM SPSS Statistics 17 (Armonk, NY, USA).

## RESULTS

Our final study population comprises 497 patients [74.6% male, mean age 51.4 (20.2) years]. The mean BSA was 2.0 (0.2 m^2^) and mean BMI 25.8 (4.8) kg/m^2^. Hypertension was the most common cardiovascular risk factor (*n* = 114, 22.9%).

### Ascending aortic and PA diameters

The mean ascending aortic diameter and length were 34.0 (4.8) mm and 83.6 (15) mm, respectively. We noted a significant difference in diameter and length in men and women. The PA diameter was 32.0 (4.6) mm [female: 31.2 (4.7) mm vs male: 32.2 (4.5) mm; *P* = 0.032]. The mean length and left and right PA diameters were similar between male and female patients. Patient baseline characteristics, cardiovascular risk factors and ascending aortic as well as PA measurements are shown in Table 2. The calculated PA diameter index was 16.6 (SD 2.7) per m^2^.

**Table 2:** Descriptive characteristics of the cohort

	Male (*n* = 371)	Female (*n* = 126)	Overall (*n* = 497)	
Age (years)	50.6 (19.6)	53.6 (21.4)	51.4 (20.2)	0.15
BSA (m^2^)	2.0 (0.2)	1.8 (0.2)	2.0 (0.2)	<0.001
BMI (kg/m^2^)	26.0 (4.4)	25.2 (5.6)	25.8 (4.8)	0.094
Dyslipidaemia	27 (7.3)	12 (9.5)	39 (7.8)	0.44
Hypertension	80 (21.6)	32 (25.4)	112 (22.5)	0.39
Smoking	58 (15.6)	16 (12.7)	74 (14.9)	0.47
Diabetes	19 (5.1)	16 (12.7)	35 (7.0)	0.008
CAD	30 (8.1)	8 (6.4)	38 (7.6)	0.70
PAH	1 (0.3)	0 (0.0)	1 (0.2)	1.00
CHD	2 (0.5)	1 (0.3)	3 (0.6)	1.00
Infective lung disease	1 (0.3)	0 (0.0)	1 (0.2)	1.00
Vasculitis	1 (0.3)	0 (0.0)	1 (0.2)	1.00
Ascending aorta diameter (mm)	34.3 (4.7)	32.8 (5.0)	34 (4.8)	0.002
Ascending aorta surface (mm^2^)	846.3 (242.0)	781.3 (250.4)	829.8 (245.5)	0.010
Ascending aorta length (mm)	84.9 (15.3)	79.8 (13.2)	83.6 (15.5)	0.001
PA diameter (mm)	32.2 (4.5)	31.2 (4.7)	32.0 (4.6)	0.032
PA surface (mm^2^)	666.2 (193.4)	640.5 (202.7)	659.7 (195.9)	0.20
PA length	49.8 (14.2)	49.4 (12.8)	49.7 (13.9)	0.78
Left PA diameter (mm)	25.0 (3.9)	24.8 (4.0)	24.9 (3.9)	0.59
Left PA surface (mm^2^)	411.0 (134.1)	407.0 (142.2)	410.0 (136.1)	0.77
Right PA diameter (mm)	25.2 (4.7)	24.9 (4.3)	25.2 (4.6)	0.51
Right PA surface (mm^2^)	425.4 (159.7)	413.3 (154.3)	422.3 (158.3)	0.46

Data are presented as number (%) or mean (standard deviation).

BMI: body mass index; BSA: body surface area; CAD: coronary artery disease; CHD: congenital heart disease; PA: pulmonary artery; PAH: pulmonary artery hypertension.

### Potential PA diameter predictors and upper limits

The stage I models of potential predicators revealed that age, BSA and smoking were significant predictors of mean pulmonary trunk diameter. The stage I model is shown in Supplementary Material, Table S1. Stage II models (included significant stage I variables and interactions) revealed BSA as only significant influencing variable for mean PA diameter. The stage II model is shown in Table 3.

**Table 3:** Stage II models: stage I models + interactions

	PA (mm)				
	*n* = 497				
	*R*^2^ = 0.12				
	Unstandardized coefficient	Standardized coefficient	Standard error	*P*-value	CI
Age	−0.02	−0.10	0.09	0.80	−0.19; 0.15
BSA	4.52	0.21	2.10	0.032	0.40; 0.64
Sum of CV risk factors	1.90	0.46	1.86	0.31	−1.74; 5.55
Smoking	−1.07	−0.08	0.71	0.13	−2.47; 0.33
Age, BSA	0.02	0.19	0.05	0.64	−0.07; 0.11
Age, sum of CV risk factors	0.02	0.34	0.01	0.13	−0.01; 0.05
BSA, sum of CV risk factors	−1.26	−0.62	0.73	0.084	−2.68; 0.17
Constant	21.80	.	3.97	<0.001	14.00; 29.59

BSA: body surface area; CI: confidence interval; CV: cardiovascular; PA: pulmonary artery.

The corresponding upper limits of the respective diameters were 41 mm (PA), 34 mm (right PA), 33 mm (left PA) and 78 mm (PA length). Detailed PA diameter stratifications by age and BSA are shown in Table 4.

**Table 4:** Pulmonary artery diameters by age and body surface area

Age	BSA (m^2^)	*n* = 497 *N*	Mean	SD	Minimum	Maximum
<45	<1.7	22	30.07	3.67	23.5	40.7
	1.7–1.9	60	29.67	3.58	22	38.1
	1.9–2.1	56	31.47	4.27	17	41.6
	≥2.1	51	32.41	3.86	25.2	41.2
45–55	<1.7	9	30.90	4.84	25.6	43.3
	1.7–1.9	16	31.85	4.49	24.7	43.8
	1.9–2.1	37	32.34	4.35	25.9	45.6
	≥2.1	23	34.45	4.25	27.8	43.2
55–65	<1.7	8	30.34	3.43	24.4	34.2
	1.7–1.9	17	29.99	4.42	22.4	38.3
	1.9–2.1	34	32.13	3.71	24.2	38.3
	≥2.1	22	32.90	4.33	27	43.4
≥65	<1.7	22	31.57	4.40	24.1	40.7
	1.7–1.9	47	33.13	5.43	20.8	44.3
	1.9–2.1	51	32.74	5.00	21.3	45
	≥2.1	21	35.30	6.22	22.1	50.6

BSA: body surface area; SD: standard deviation.

Supplementary Material, Table S2 shows left PA diameter stratifications by age and BSA. Right PA and PA length stratifications are summarized in Supplementary Material, Tables S3 and S4.

Using these values, regular mean PA diameter can be calculated according to the patient’s individual risk profile. The formula for predicting the mean PA diameter in patients younger than 45 years of age is 4.31 × BSA + 22.56, in patients aged 45–54 years is 4.7 × BSA + 23.37, 55–64 years is 5.71 × BSA + 20.43 and older than 65 years of age is 4.6 × BSA + 24.27, which means that one only has to calculate the BSA according to the patient’s weight and height and insert this value in the equation of the respective age category.

We have created an online calculator to calculate the normal diameter of the PA diameter based on age, height, weight, sum of cardiovascular risk factors and smoking history (www.pa-diameter.com).

**Figure 2:**
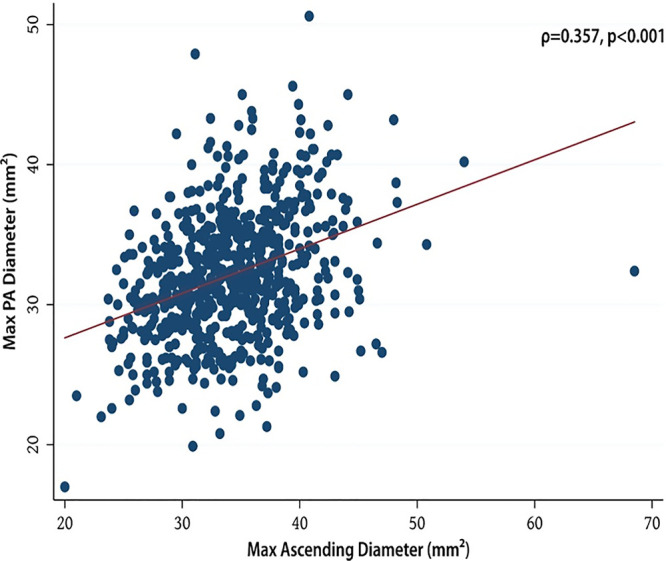
Correlation between pulmonary artery and mid-ascending diameter.

Figure 2 shows the significant correlation (*r* = 0.352; *P* < 0.001) between PA and the mid-ascending aortic diameter.

## DISCUSSION

This study’s 3 most essential findings can be summarized as: (i) normal mean PA diameter in a representative patient cohort (primarily non-cardiovascular patients) is 32.0 ± 4.6 mm; (ii) BSA is the only influencing variable of PA diameter; and (iii) measured normal diameters and the corresponding upper limits of normal, as well as the previous definition of arterial aneurysms (1.5-fold the normal diameter) revealed that our threshold’s definition for PAA should not be <45 mm.

Few studies have investigated the issue of PAA or discussed what constitutes normal diameters of the main, right and left PAs and their correlation with the ascending aorta’s diameter. The current definitions mainly rely on postmortem data from Deterling and Clagett in 109 571 cadavers published 1947 [15] and the Framingham Heart Study’s 3171 patients [6]. In the latter, only native CT scans were analysed in only one axial (two-dimensional) slice. Data from the Framingham Heart Study revealed a mean diameter of 25.1 ± 2.8 mm. These obviously deviate substantially from our results (32.0 ± 4.6 mm), a fact attributable to (i) the quite out-dated postmortem measurements with all their problems concerning transferability to the present day and (ii) our study’s more precise measurement methods compared to those taken by Truong *et al.* [6]. What is considered a pathological PAA has very different definitions, i.e. from >29 mm (exceeding normal diameter) [9], over 40 mm [8] and >1.5 times more than a poorly defined normal value [10] with gender-specific differences of 3 mm (43.4 vs 40.4 mm) taken from Framingham Heart Study data. According to our measurements, namely a 32.0 ± 4.6-mm mean diameter and 41-mm upper limit of normal (and in line with the literature), we recommend that the limit not be <45 mm to define a PAA. We propose a PA diameter of not less than 45 mm as a result of the calculated normal’s upper limit and 1.5-fold more than normal diameter (the normal’s upper limit + 1.5-fold the normal diameter/2). This diameter cutoff may well apply to both genders and all age groups, since neither gender nor age have revealed clinically relevant differences in absolute diameters. In patients presenting diameters measuring between the normal and the limit, using the term *ectasia* may be appropriate.

Previous studies have identified the thoracic aorta’s normal diameter and predictors of an increasing aortic diameter (age, BSA, gender and hypertension) [14]. The fact that the BSA has been identified as an influencing variable of thoracic vessel diameters, as our study also shows for PA diameters, appears conclusive. In this regard, it makes sense that we identified a significant correlation between the PA’s diameter maximum and diameter of the ascending aorta. Nonetheless, other cardiovascular risk factors, as with thoracic aortic diameters, appear to play a minor role in determining PA size. Of note, patients with potentially contributing cardiac and structural pulmonary disease were excluded from analyses which may be the reason that PAH was not found as predictor for PA size.

While this study helps considerably to specifically define a PAA, 2 questions remain regarding a possible diameter threshold for interventions and how a PAA should be treated. There seems to be general consensus in the literature for smaller PAAs to be medically managed conservatively, since PAA-associated complications such as dissection, rupture or local displacement of surrounding structures occur seldom and are more likely to occur in PAAs of larger diameter [10]. Medical treatment should be tailored to the underlying disease, co-morbidities and the presence of PAH and should include close follow-up and possibly oral anticoagulation. We have recommended surgical interventions in PAAs larger than 55 mm, increase in diameter of >5 mm within 6 months, compression of adjacent structures, thrombus formation in the aneurysm sack (despite oral anticoagulation), persisting clinical symptoms not resolving after medical therapy, evidence of valvular pathologies or shunt flow and/or signs of rupture or dissection [5].

**Figure 3:**
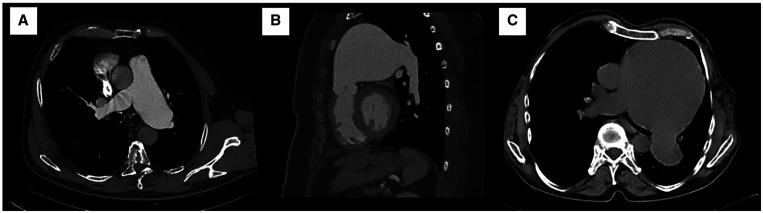
Preoperative computer tomography of a patient with a left pulmonary artery aneurysm of 58 mm (**A**), a pulmonary artery aneurysm of 76 mm (**B**) and 105 mm (**C**).

**Figure 4:**
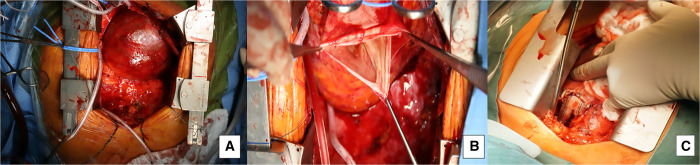
Perioperative view of a giant pulmonary artery aneurysm before (**A**) and after (**B**) opening the aneurysmal sac with a very thin pulmonary artery wall. (**C**) Intraoperative view of a reversed Y-prosthesis in place after left and right pulmonary artery resection.

There are various surgical treatment options proposed in the literature: the 2 main surgical strategies are aneurysmorraphy and PAA resection and replacement [13, 16]. Aneurysmorrhaphy is a relatively rapid, effective procedure for reducing PA diameter with good clinical outcomes [16]. However, the risk of aneurysm recurrence remains unclear, as the procedure merely reduces the vessel’s diameter but does not treat the abnormal vessel wall or reduce the overall wall stress according to the law of Laplace. Thus, aneurysm resection and replacement seems to be the method of choice, a technique that entails various surgical options. The simplest option is to replace the entire PA using a Dacron prosthesis and to perform distal anastomosis before the pulmonary bifurcation to treat a genuine main PAA. If necessary, the pulmonary valve can be spared via supravalvular PAA resection, repaired by valve-sparing techniques (‘pulmonary David procedure’), or replaced if the valve is structurally damaged [17, 18]. If the left and right PA are also aneurysmatic (Fig. 3) and the distal anastomosis cannot be done before the pulmonary bifurcation, a reversed Y-prosthesis (normally used for infrarenal aortic replacement) can be anastomosed proximally to the left and right PA origin (Fig. 4). If the right and left PA’s diameter exceeds the limit for a reversed Y-prosthesis, we connect the right and left PA via a Dacron graft and generate a T-graft to the main PA afterwards (Video 1). In case of a PAA of infectious origin, replacement with xenopericardial tube grafts may be feasible to reduce the risk of re-infection [19, 20]. Of note, all the surgical options available suffice for primary repair, but there are no data that clearly favors any one of these different surgical strategies. Moreover, we wish to stress that performing the distal suture line may be complicated by the PA’s extremely thin wall, as Fig. 4 shows.

### Limitations and strengths

This is a retrospective, single-centre study with all their inherent limitations. We have focused on 1 specific patient collective and cannot rule out a possible selection bias regarding the PA diameter, nor can we exclude other possible influencing factors on PA diameter. Therefore, BSA should be interpreted and applied cautiously in very small or obese patients. Our diameters were measured much more precisely, and in a sufficient number of patients than in other studies. Moreover, there is an absence of model fit criteria as part of the model building steps in our exploratory approach and the treatment of missing data and data quality may be another limitation.

## CONCLUSIONS

Assessing PA diameter by taking three-dimensional centreline measurements in a representative patient cohort after contrast-enhanced, whole-body CT is a very precise method by which to define normal PA diameters. Normal mean PA diameter is 32.0 ± 4.6 mm. BSA proved to be the only influencing variable of pulmonary trunk diameter. The normal diameters and corresponding upper limits of normal measured in this study reveal that our definition of the PAA’s threshold should not be <45 mm. We also discussed the indications and surgical treatment options for PAA that effectively reduce the risk of subsequent complications.

## SUPPLEMENTARY MATERIAL

Supplementary material is available at *ICVTS* online.

## Funding

This study receives institutional funding. 

**Conflict of interest:** none declared.

### Author contributions

**Tim Berger:** Conceptualization; Validation; Writing—original draft. **Matthias Siepe:** Conceptualization; Supervision; Visualization; Writing—review & editing. **Björn Simon:** Conceptualization; Data curation; Formal analysis; Visualization; Writing—review & editing. **Friedhelm Beyersdorf:** Supervision; Validation; Writing—review & editing. **Zehang Chen:** Formal analysis; Methodology; Visualization; Writing—review & editing. **Stoyan Kondov:** Data curation; Software; Visualization; Writing—review & editing. **Christopher L. Schlett:** Conceptualization; Supervision; Writing—review & editing. **Fabian Bamberg:** Supervision; Writing—review & editing. **Aleksandre Tarkhnishvili:** Data curation; Writing—review & editing. **Salome Chikvatia:** Data curation; Writing—review & editing. **Martin Czerny:** Visualization; Writing—review & editing. **Bartosz Rylski:** Conceptualization; Methodology; Supervision; Writing—review & editing. **Maximilian Kreibich:** Conceptualization.

### Reviewer information

Interactive CardioVascular and Thoracic Surgery thanks Diego Alejandro Murillo Brito, Mateo Marin-Cuartas and the other, anonymous reviewer(s) for their contribution to the peer review process of this article.

## Supplementary Material

ivab308_Supplementary_Data

## ABBREVIATIONS

BSA: Body surface area
CT: Computed tomography
PA: Pulmonary artery
PAA: Pulmonary artery aneurysm
PAH: Pulmonary artery hypertension
SD: Standard deviation
