# Supplementary material for: Pulmonary artery diameter: means and normal limits—assessment by computed tomography angiography
Source: Interact Cardiovasc Thorac Surg. 2021 Nov 17;34(4):637–44. doi: 10.1093/icvts/ivab308 (PMC9026207; doi:10.1093/icvts/ivab308)
Supplement: ivab308_Supplementary_Data [file ivab308_Supplementary_Data.pdf]

Supplemental material

Supplemental Table 1. Stage I: Potentially clinical predictors of PA diameter

	**n =**	**497**			
	R^2 =	0,11			
**Predictor**	**Unstandardized Coefficient**	**Standardized Coefficient**	**Standard-Error**	***P value***	**CI**
Age	0.03	0.15	0.01	0.004	0.01; 0.06
BSA	5.23	0.25	1.59	0.001	2.12; 8.35
BMI	-0.12	-0.13	0.06	0.057	-0.25; 0.00
Sum of CV risk factors	1.83	0.44	0.93	0.050	0.00; 3.66
Sex	0.03	0.00	0.61	0.96	-1.17; 1.24
Smoking	-2.42	-0.19	1.09	0.026	-4.56; -0.29
Diabetes	-1.45	-0.08	1.52	0.34	-4.44; 1.54
Dyslipidemia	-0.94	-0.06	1.23	0.44	-3.37; 1.48
Hypertension	-0.92	-0.08	1.15	0.43	-3.18; 1.35
CAD	-1.49	-0.09	1.45	0.30	-4.34; 1.35
Constant	22.76		2.17	<0.001	18.49; 27.03

BSA, body surface area (predicted increase in mm PA diameter per 1m^2 increase in BSA); BMI, body mass index; CI, confidence interval; CV cardiovascular

Supplemental Table 2 left PA diameters by Age and BSA

		Left PA Diameter (mm^2) n = 497 (total = 497, deleted = 0)				
**Age**	**BSA (m^2)**	**N**	**Mean**	**Sd**	**Min**	**Max**
<45	<1.7	22	21.67	2.26	17.3	25.3
	1.7-1.9	60	21.73	2.71	12.5	28.2
	1.9-2.1	56	22.83	3.06	14	32.2
	≥2.1	51	23.10	2.53	18	30.1
45-55	<1.7	9	24.21	6.43	18.8	40.3
	1.7-1.9	16	24.68	3.19	16.5	29.4
	1.9-2.1	37	24.61	2.81	19.3	31.8
	≥2.1	23	25.48	3.12	19.5	31.8
55-65	<1.7	9	22.89	2.08	20.5	25.8
	1.7-1.9	17	27.25	2.34	21.8	31.4
	1.9-2.1	34	25.48	3.26	20.2	33
	≥2.1	22	26.37	3.33	22	33.6
>=65	<1.7	22	26.65	3.73	20.6	32.8
	1.7-1.9	47	28.17	3.36	22.2	35.4
	1.9-2.1	51	27.89	3.72	22.8	37.9
	≥2.1	21	28.68	3.68	21.6	37.3

PA, pulmonary artery; BSA, body surface area; sd, standard deviation

Supplemental Table 3. Right PA diameters by Age and BSA

		Right PA Diameter (mm^2) n = 497 (total = 497, deleted = 0)				
**Age**	**BSA (m^2)**	**N**	**Mean**	**Sd**	**Min**	**Max**
<45	<1.7	22	21.92	2.63	16.8	28.9
	1.7-1.9	60	22.11	2.57	17.8	28.2
	1.9-2.1	56	22.56	2.94	16	27.9
	≥2.1	51	22.28	3.22	15	30.5
45-55	<1.7	9	21.52	1.86	18.9	24.3
	1.7-1.9	16	25.58	3.74	18.3	32.2
	1.9-2.1	37	25.02	5.39	18.9	52.1
	≥2.1	23	25.21	3.00	20.7	31.6
55-65	<1.7	9	23.10	3.06	17.8	26.5
	1.7-1.9	17	26.85	3.09	17.9	29.8
	1.9-2.1	34	25.62	3.04	19.8	31.3
	≥2.1	22	27.58	3.11	22.8	33.7
>=65	<1.7	22	27.14	3.18	20.3	32.6
	1.7-1.9	47	29.00	4.88	19.4	40.1
	1.9-2.1	51	28.71	4.64	20.8	38.3
	≥2.1	21	29.70	4.88	22.4	39.7

PA, pulmonary artery; BSA, body surface area; sd, standard deviation

Supplemental Table 4. PA length by Age and BSA

		Max PA Length (mm)				
		n = 497	(total = 497, deleted = 0)			
**Age**	**BSA (m^2)**	**N**	**Mean**	**Sd**	**Min**	**Max**
<45	<1.7	22	45.33	10.83	22.8	68.2
	1.7-1.9	60	44.82	11.23	22.6	74
	1.9-2.1	56	44.08	12.95	17.2	70.4
	≥2.1	51	48.36	11.00	19.2	63.6
45-55	<1.7	9	42.04	12.20	26.9	57.7
	1.7-1.9	16	47.09	13.61	18.4	64.6
	1.9-2.1	37	48.08	13.61	23.8	70.8
	≥2.1	23	55.49	13.35	28.8	76.1
55-65	<1.7	9	42.42	14.37	23	63.5
	1.7-1.9	17	51.66	9.55	34.9	67.9
	1.9-2.1	34	50.63	14.48	26	73.2
	≥2.1	22	57.20	17.17	26.4	96.7
>=65	<1.7	22	50.61	11.75	27.9	67.2
	1.7-1.9	47	53.93	15.58	20.8	75.8
	1.9-2.1	51	54.51	14.48	23.5	89
	≥2.1	21	57.43	15.00	24.3	88.8

PA, pulmonary artery; BSA, body surface area; sd, standard deviation

Supplemental figure 1.

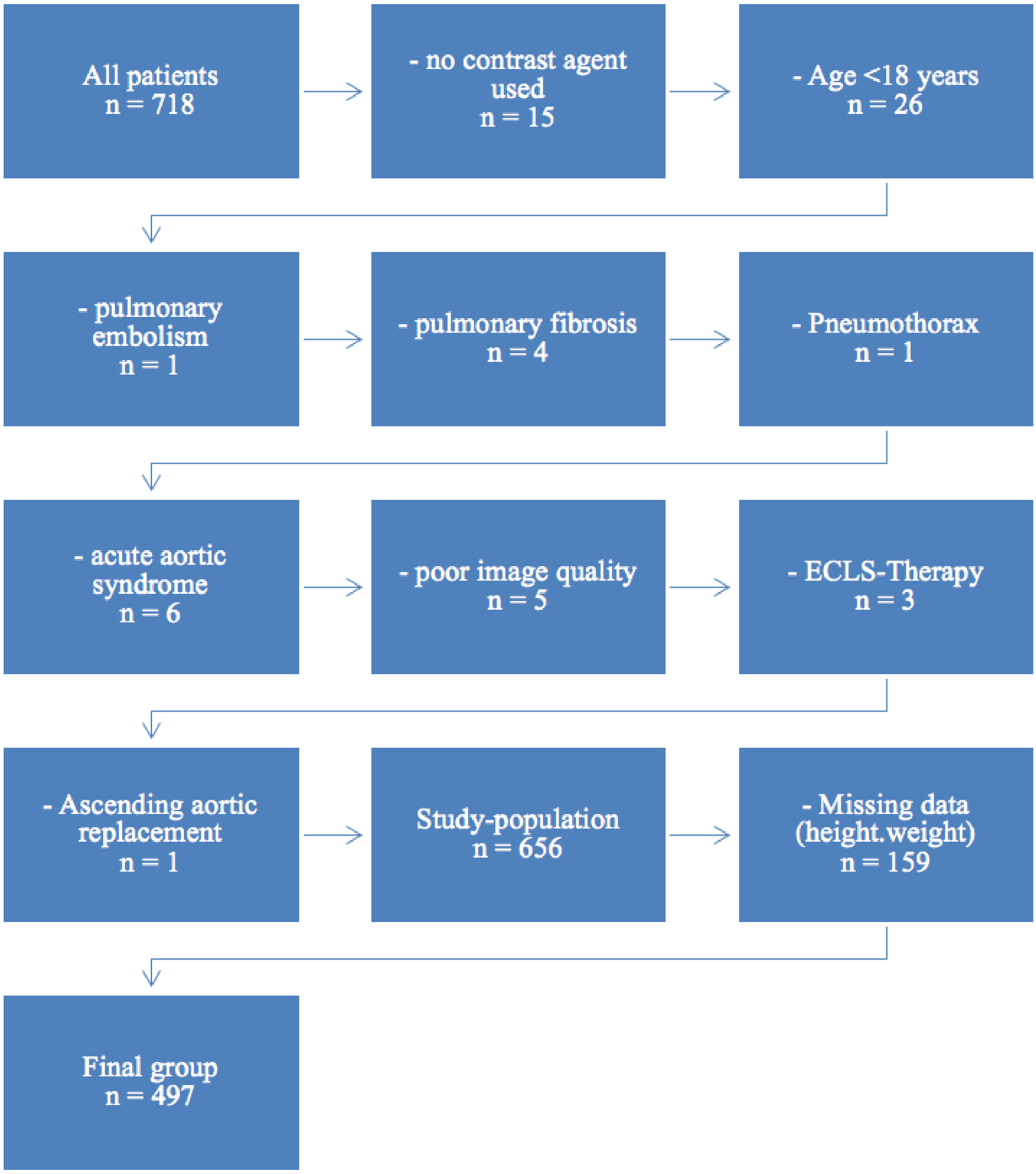

Initial patient cohort and exclusion criteria to create the final study group.
